# The use of deep learning integrating image recognition in language analysis technology in secondary school education

**DOI:** 10.1038/s41598-024-52592-5

**Published:** 2024-02-05

**Authors:** Liqing Chu, Yanlan Liu, Yixi Zhai, Dandan Wang, Yufei Wu

**Affiliations:** School of Foreign Studies, Tangshan Normal University, Tangshan City, 063000 China

**Keywords:** Materials science, Mathematics and computing

## Abstract

This work aims to investigate the application of advanced deep learning algorithms and image recognition technologies to enhance language analysis tools in secondary education, with the goal of providing educators with more effective resources and support. Based on artificial intelligence, this work integrates data mining techniques related to deep learning to analyze and study language behavior in secondary school education. Initially, a framework for analyzing language behavior in secondary school education is constructed. This involves evaluating the current state of language behavior, establishing a framework based on evaluation comments, and defining indicators for analyzing language behavior in online secondary school education. Subsequently, data mining technology and image and character recognition technology are employed to conduct data mining for online courses in secondary schools, encompassing the processing of teaching video images and character recognition. Finally, an experiment is designed to validate the proposed framework for analyzing language behavior in secondary school education. The results indicate specific differences among the grouped evaluation scores for each analysis indicator. The significance p values for the online classroom discourse’s speaking rate, speech intelligibility, average sentence length, and content similarity are −0.56, −0.71, −0.71, and −0.74, respectively. The aim is to identify the most effective teaching behaviors for learners and enhance the support for online course instruction.

## Introduction

The swift evolution of artificial intelligence (AI) technology has garnered considerable attention for its application in secondary education. Notably, language analysis technology, an integral facet of AI, holds substantial promise within the realm of secondary education. This study seeks to assess the efficacy of AI-based language analysis technology in secondary education, aiming to furnish a scientific foundation for educational reform. Technological innovations are reshaping secondary education as online education gains popularity and evolves. Language analysis technology, leveraging techniques like natural language processing and text analysis, can delve into students’ linguistic expressions during the learning process, thereby equipping educators with a more comprehensive understanding of students’ learning dynamics. Through AI, a nuanced analysis of students’ language proficiency, expression patterns, and related aspects becomes feasible, offering precise guidance for personalized teaching and subject-specific tutoring.

In the online environment, teaching behavior can significantly impact learners’ experiences and learning outcomes. Therefore, as a crucial dimension of teaching practice, teaching behavior plays a pivotal role in influencing the effectiveness of instruction. Studying this controlling mechanism can help promote online courses and facilitate more efficient student learning^1^. Some scholars have found a significant correlation between teaching behavior and academic emotion, arguing that teaching behavior can alleviate students’ negative emotions online, such as anxiety and loneliness^2^. Conversely, online teaching behavior serves as a direct expression of educators’ teaching abilities and comprehensive skills. Educators must reflect on their teaching behaviors to enhance the effectiveness of online instruction. Therefore, the foundation for building high-quality online courses should begin with the online Teaching Behavior Analysis (TBA)^3^.

Based on the media used by educators, teaching behaviors can be categorized into verbal and non-verbal behaviors. Notably, classroom discourse is fundamental for student–teacher communication, constituting approximately 80% of all teaching behaviors^4^. Makarenko, a renowned educator in the former Soviet Union, emphasized that, under the same teaching model, different classroom discourses might lead to a 200-fold difference in teaching effectiveness, underscoring the importance of classroom discourse^5^. Additionally, classroom discourse, a crucial component of educators’ teaching behavior, serves as a key indicator in evaluating the quality of online courses^6^. Therefore, focusing on online TBA and leveraging big data technologies to mine its characteristics and patterns holds great significance for enhancing the teaching quality and learning outcomes of online courses^7^.

The innovative development of online course-supportive big data platforms and related data processing technologies has become a new research focus. Understanding how classroom discourse influences the learning experience and teaching effectiveness is essential to improve online educators’ essential teaching skills. To this end, this work introduces big data mining technology to explore educators’ teaching characteristics and behaviors that affect the quality of online courses. It analyzes the teaching objectives, evaluates online educators’ experiences, and explores online TBA methods. Based on the research findings, implications are suggested for enhancing online educators’ teaching skills. The research results provide an essential reference and basis for improving the online learning experience and teaching effectiveness.

## Literature review

The online course-oriented data mining technology based on AI targets the unique data collected from the teaching environment, teaching objects, and teaching process in online courses. It focuses on big data in online courses, which falls into the main category of educational big data research and application^8^. Recently, the application and research of educational data mining technology in online courses have been increasing. For example, Ma et al. (2022) used clustering algorithms in data mining technology to analyze online learning data, group them with similar learning characteristics, and assess students’ progress^9^. Based on college students’ data, Varade and Thankanchan (2021) employed a decision tree algorithm to explore the factors influencing students’ success, introducing a new educational data mining architecture^10^. Yulianci et al. (2021) analyzed the behavioral characteristics of 2,801 online learners and explored the relationship between subjects’ learning effects and the online learning system^11^. Ko et al. (2021) used logistic regression to model data from three Massive Open Online Courses (MOOCs) in America, providing suggestions for improving the quality of MOOC teaching^12^.

Scholars have extensively researched educational data mining, online courses, online course teaching quality, educators’ teaching characteristics, and TBA, both theoretically and practically. There is a research gap in secondary school education-oriented classroom discourse analysis (CDA). Notably, the particularity of teaching methods in the secondary school teaching environment has been considered in sporadic cases. However, their research focuses on the expressive skills and techniques of classroom discourse, providing a reference for this work. Meanwhile, some problems are noticeable in the existing research. For example, the analysis is not systematic enough, the source of evaluation indicators is unclear, and there is no further in-depth analysis and research on various indicators. Based on this, this work addresses a current research gap by comprehensively analyzing discourse within secondary school-oriented classrooms. Focusing on the unique characteristics of the secondary school teaching environment, the present work explores the expressive features of classroom discourse and its correlation with teaching effectiveness. The work integrates AI-based technologies with the educational data mining approach to conduct a meticulous analysis of classroom discourse. The objective is to offer scientifically grounded improvement recommendations for online secondary education, thereby positively contributing to the enhancement of teaching quality and student learning outcomes. This work introduces novel perspectives and methodologies to the field of secondary education, fostering the advancement of online education. Furthermore, it extends the application of educational data mining technology within secondary school teaching practices.

## Construction of the CDA framework in secondary school education

### Current situation

With the informatization, networking, and intelligence in education, various secondary school education models have emerged, such as online courses, flipped classes, and mixed teaching. The rapid development of educational data mining and educational intelligence technology has brought new opportunities for TBA, including CDA. Consequently, the importance of classroom discourse in secondary school education has been greatly emphasized^13,14^. Theoretical analysis and empirical tests suggest that classroom discourse is directly related to the dissemination effect of teaching information. The value of classroom discourse is reflected in stimulating students’ positive emotions and positioning them as autonomous, meta-reflective, and communicative learners. The language expression skills of educators will impact the learning mood and learning effect. Coordinating the use of vocal and non-vocal discourse can help transmit educational content and skills more clearly to learners over the Internet while overcoming spatial–temporal constraints^15^.

### Construction of CDA framework based on evaluation perspective

Building upon the analysis of typical CDA methods discussed earlier, online classroom discourses in secondary schools can be categorized into two dimensions: basic features and strategic features. Basic features pertain to the inertial behavior of educators when engaging in discourse without thoughtful consideration or reprocessing^16^. In contrast, strategic features are more intentional, where educators personalize and design teaching content using specific discourse based on their teaching experience and current objectives. The teaching content is then delivered through logically organized discourse^17^.

**Figure 1:**
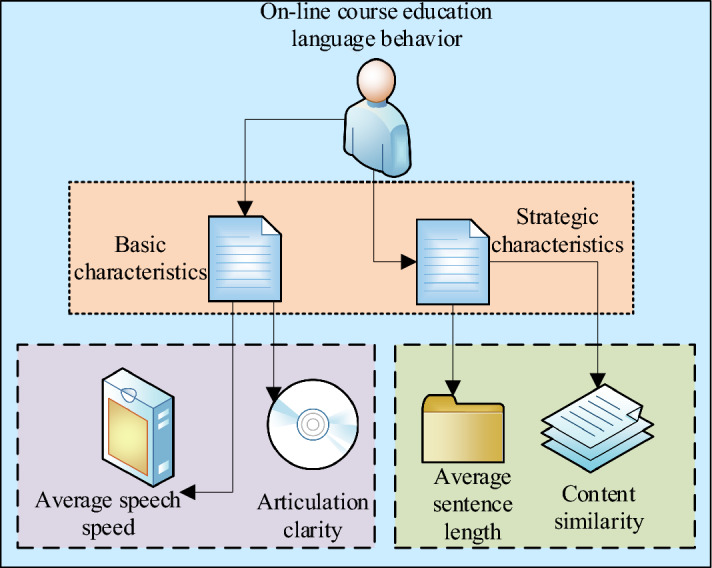
Framework of online CDA in secondary schools.

Consequently, this work organizes the CDA framework for online education in secondary schools into two dimensions: basic features and strategic features, as illustrated in Fig. 1.

Figure 1 illustrates that basic feature dimensions primarily revolve around two key indicators: speech rate and speech clarity. These indicators play a crucial role in assessing speech skills and expressive abilities. Speech rate gauges the speaker’s language generation proficiency within a unit of time, while speech clarity measures pronunciation accuracy and intelligibility. At a foundational level, these two dimensions serve as primary criteria for evaluating speech expression quality. Conversely, the evaluation of strategic feature dimensions places more emphasis on average sentence length and content similarity. Average sentence length reflects the speaker’s approach to expressing viewpoints or information; shorter sentences may convey concise information, while longer sentences may provide more detailed expressions. Content similarity assesses the consistency and thematic relevance of speech expression, determining whether the speaker maintains logical and thematic coherence. A comprehensive assessment of these feature dimensions thoroughly explains speech expression quality and characteristics. When formulating improvement strategies or enhancing speech expression, adjustments in aspects such as speech rate, speech clarity, sentence length, and content similarity can be selectively made to achieve superior expressive outcomes. This multidimensional assessment approach contributes to the comprehensive enhancement of overall speech expression, meeting diverse expressive requirements across various scenarios and needs.

### Construction of online CDA indicators for secondary schools

#### Speaking rate of online classroom discourse

The speaking rate can be explicitly understood as the number of words or syllables per unit of time. The statistical unit for speaking rate in Chinese is generally expressed as Words Per Minute (WPM)^18^. This work defines speaking rate as the average speed at which educators talk throughout a class, with pauses between sentences also considered in the calculation^19^.

Analysis of learner’s evaluation comments reveals that learners often focus on educators’ speaking rates when evaluating online courses for secondary education. The comments are categorized in Table 1.

**Table 1:** Evaluation comments on the educators’ speaking rate in the online classroom of the secondary schools.

Category	Number	Evaluation comments
Positive comments	1	The explanation was very detailed, moderate, and easy to understand
	2	The teacher explained the teaching content step by step, maintaining a moderate pace that stimulates thought
	3	The course was well-organized, featuring beautifully produced videos with clear explanations
Negative comments	1	The teacher read the text only once and did so quickly
	2	Perhaps the teacher spoke too fast, resulting in efficiency not being as good as offline
	3	The teacher spoke too fast when explaining specific key points, making students unable to hear clearly

The speaking rate has a significant impact on online learning. Some educators speak too fast, making it challenging for learners to keep up, while others might talk too slowly, affecting learning interest and garnering negative comments. Therefore, setting a fixed standard for the speaking rate in online courses is challenging. This work assumes that the average speaking rate should fall within a specific range, referencing most existing research. In general, a slower speaking rate can aid online learners in better understanding and learning than a faster speaking rate.

#### Speech intelligibility of online classroom discourse

Speech intelligibility refers to the degree to which the speaker’s words are understood by the listener and is influenced by factors such as voice construction, speed, fluency, quality, and intensity^20,21^.

Similarly, an analysis of online evaluation comments reveals that learners often consider the standard level of pronunciation and the clarity of pronunciation and intonation. Some evaluation comments are categorized in Table 2.

**Table 2:** Selected evaluation comments on speech intelligibility of online courses in secondary schools.

Category	Number	Evaluation comments
Positive comments	1	The teacher’s Mandarin pronunciation is clear and standard, and I can listen comfortably without watching subtitles
	2	The teacher’s Mandarin is very standard; I like it very much
	3	The teacher’s language intonation sounds very friendly and is easy to understand
Negative comments	1	The teaching is average, and the speaker’s Mandarin needs to be improved
	2	The teacher’s guidance is good, but his/her Mandarin is not standard. Many key points are unclear
	3	The speaker’s proficiency in Mandarin requires enhancement. While it does not hinder student comprehension, it diminishes the overall listening experience

Based on Table 2, this work selects Mandarin clarity as an evaluation indicator for CDA in online courses, serving as a fundamental feature of classroom discourse.

#### Average sentence length of classroom discourse in online courses

Sentence length is a crucial indicator of sentence complexity^22^. Relevant research points out that sentence length significantly impacts learners’ understanding. Long sentences, especially in language teaching, can pose challenges for students^23^. Analysis of learners’ online evaluation comments indicates their emphasis on language organization. Excellent language organization is often evaluated as concise, to the point, clear, simple, logically structured, and easy to understand^24^. Many of these comments are linked to the impact of classroom discourse on the cognitive load of teaching objects. The complexity of classroom discourse can be measured by the length of sentences spoken. Some evaluation comments are categorized in Table 3.

**Table 3:** Part of the evaluation comments on classroom discourse complexity in online courses for secondary schools.

Category	Number	Evaluation comments
Positive comments	1	The teaching content was systematically organized, and the language used was concise with a strong rhythmic sense
	2	The teaching language was logically clear and concise
	3	The teaching content was straightforward, and the courseware was simple and clear
Negative comments	1	The teaching language was abstract, with the teacher mainly mechanically reading the courseware
	2	The teacher complicated simple questions, making understanding difficult for students
	3	The teaching explanations are repetitive and lack logic, disappointing in language expression

As shown in Table 3, the length of sentences is a fundamental indicator for evaluating the complexity of classroom discourse. Therefore, this work designates average sentence length as one of the strategic features of classroom discourse in online education for secondary schools.

#### Content similarity of classroom discourse in online courses

In today’s teaching practice, there are often phenomena, such as reading from books and leaving textbooks, which seriously affect the improvement of teaching quality. The degree to which the teachers read or mechanically copy the textbook or courseware is defined as content similarity^25^. Further mining learners’ evaluation comments implies that most learners strongly oppose the high content-similar teaching behaviors, such as reading books or reading courseware. Some evaluation comments are classified in Table 4.

**Table 4:** Partial evaluation comments from learners on teacher explanations of texts.

Category	Number	Evaluation comments
Positive comments	1	The teacher avoids mechanical repetition of courseware. The content is straightforward and easy to understand
	2	The course is engaging without rigidly reading courseware content
	3	Examples align well with textbook knowledge explanations
Negative comments	1	Teaching followed the text in a rigid and formalized manner, lacking liveliness
	2	Lecture knowledge is comprehensive, but the content lacks vibrancy
	3	The content explanation is flat and dull

Based on this, the present work designates content similarity of online courses as one of the strategic features of classroom discourse in secondary schools.

## Analysis and application of CDA in secondary school online courses based on AI

### Video data mining of online courses based on AI

#### Video resource identification technology

Video is a multimedia resource combining visual and auditory elements, with the teaching video carrying the main instructional content of the course. Notably, classroom discourse primarily transmits through the auditory channel. Therefore, recognizing speech in the teaching video allows for the extraction of semi-structured classroom discourse text. On the other hand, teaching courseware (teaching content) is predominantly conveyed through the visual channel. Hence, recognizing text from the images in the teaching video enables the extraction of semi-structured teaching courseware text^26^.

(1) Speech recognition of video

Speech recognition, also known as Automatic Speech Recognition (ASR), is a Human–Computer Interaction method that converts unstructured audio stream data into semi-structured text. It facilitates machine understanding and generates corresponding operations, ultimately achieving hidden information mining in speech^27^.

(2) Character recognition of video image

Computer vision aims to understand images, and recognizing characters from images is commonly referred to as Optical Character Recognition (OCR). This work opts for OCR to obtain semi-structured teaching courseware text by recognizing the images in the teaching video. The text recognition process used here involves high-level semantic logic analysis. Moreover, existing OCR technology is relatively mature, with Baidu AI Cloud’s OCR module demonstrating high accuracy in general scene character recognition. Thus, it provides a solid technical foundation for extracting characters from teaching video images and obtaining teaching content in this work^28^.

#### Text similarity measurement

Text similarity is a pivotal indicator for information retrieval, document detection, and text mining. It gauges the differences and commonalities between texts with basic calculation methods, including string matching and word matching. As Natural Language Processing (NLP) technology has progressed, additional methods such as stem extraction, stop word removal, and part-of-speech tagging have been integrated into Text Similarity Measurement (TSM). Contemporary TSM methods often combine semantic information with various weighting, regularization strategies, and NLP techniques^29^.

Building upon the preceding analysis, this work aims to employ the TSM method to assess the similarity between the classroom discourse of online courses in secondary schools and the teaching courseware. Subsequently, it analyzes the disparities and similarities between the two, evaluates the degree of “Scripted Teaching” by teachers, and offers insights for enhancing classroom discourse.

### Design of classroom discourse calculation process for online education in secondary schools

#### Ideas of classroom discourse calculation in the online course

**Figure 2:**
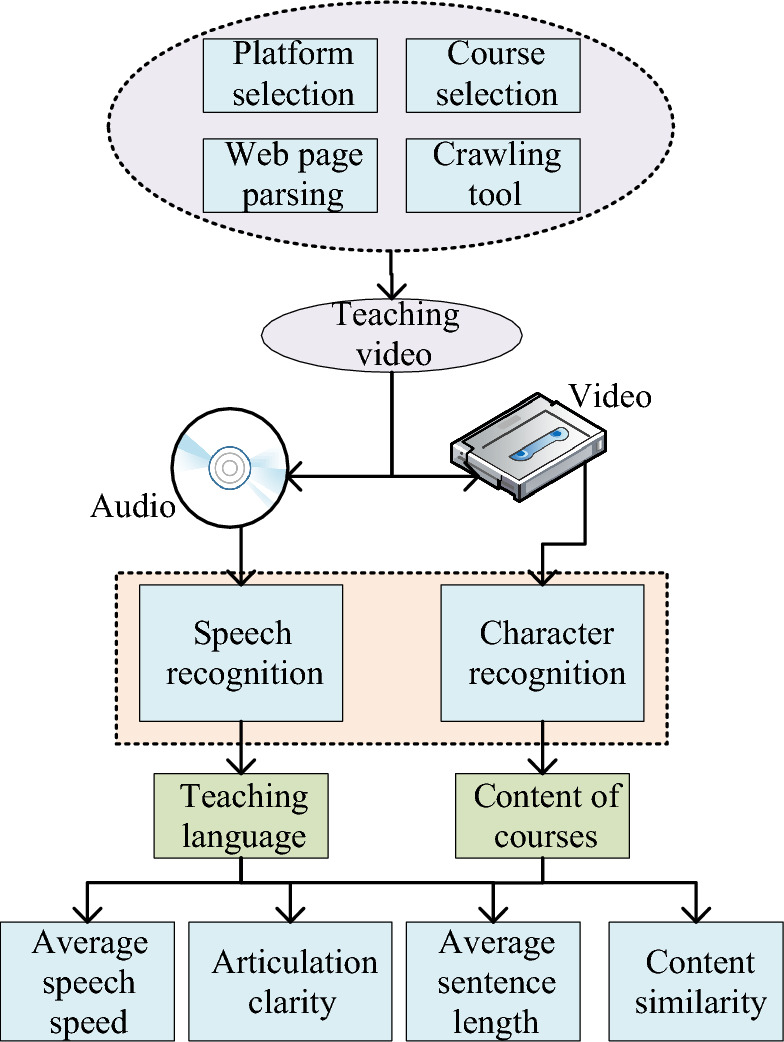
Ideas on the calculation of classroom discourse indicators of the online classroom in middle schools.

Drawing from the theoretical foundation of the analysis framework for classroom discourse in online courses for secondary schools, a specific experiment is conducted from the perspective of the teaching object. This involves using the online course teaching video as the research subject and employing data crawler technology to acquire educational data. Simultaneously, intelligent technologies and techniques such as ASR, text recognition, and TSM are applied to transform unstructured teaching videos into semi-structured text data. This approach explores a scientifically sound method for calculating and analyzing the four indicators of online classroom discourse. The structured calculation of these four indicators is realized, as depicted in Fig. 2.

Figure 2 illustrates this work’s focus within the discourse analysis framework in secondary school online courses, emphasizing four key indicators: clarity of speech expression, average sentence length, content similarity, and average speech rate. Clarity, as a pivotal indicator, gauges the accuracy and comprehensibility of the speaker’s pronunciation, constituting a fundamental element in evaluating the quality of speech expression. Examining average sentence length offers insights into how educators convey viewpoints or information, revealing the expressive style and structure of classroom discourse. Content similarity assesses speech expression’s consistency and thematic relevance, probing whether the speaker maintains logical and thematic coherence in the classroom. Lastly, average speech rate measures the speed at which the speaker generates language in a unit of time, a critical factor for effective communication and student comprehension in online education scenarios. The comprehensive analysis of these four indicators ensures robust support for the scientific computation and in-depth understanding of discourse in online classrooms.

#### Online classroom discourse calculation process

(1) Online course video acquisition and format conversion

Initially, each major online course platform is chosen as the data platform for analyzing secondary school courses. The platform crawler protocol is analyzed, and the crawler program is employed to obtain teaching video resources. Subsequently, the format of the collected video resource set is converted, and audio resources containing classroom discourse and image resources displaying courseware content in the video are obtained. These data lay the foundation for subsequent analysis and calculation.

(2) Converting the classroom discourse and teaching content into text

Python is utilized to call the Alibaba Cloud intelligent speech recognition interface and Baidu AI Cloud general scene text interface, enabling speech recognition for audio resources containing educational language behavior. Following this, text recognition is performed for image resources containing courseware content. This process extracts the text format of classroom discourse from audio files and the text format of teaching content from images. Finally, semi-structured data text is obtained for further analysis and calculation.

(3) Parameter calculation of various indicators of classroom discourse

This experiment is conducted based on the theoretical foundation of the CDA framework for online courses in secondary schools from the teaching object’s perspective. Considering relevant research and the analysis of the current situation, four indicators for online classroom discourse are designed: speaking rate, speech intelligibility, average sentence length, and text similarity. These indicators transform unstructured text resources into a structured quantity format.

### Experimental design

#### Experimental data

To efficiently analyze the online classroom discourse in secondary schools, experimental data are gathered from major online education network platforms. The video data of the secondary school online curriculum is acquired using the data crawler method. Subsequently, based on the foundational data, including evaluation comments, teaching videos, and other resources in the online curriculum, data mining, format conversion, and numerical calculations are performed. This process yields various data points such as speaking rate, speech intelligibility, average sentence length, and content similarity. The collected dataset serves as samples for testing research hypotheses.

#### Data analysis

Next, the Statistical Package for the Social Sciences (SPSS) is utilized to conduct descriptive statistics, variance analysis, and regression analysis on the acquired data samples. The results aim to reveal the impact of online classroom discourse on course grading.

## Testing the effect of CDA of the online course in secondary schools

### Analysis of inter-group differences in classroom discourse and course grading

#### Descriptive statistics

**Figure 3:**
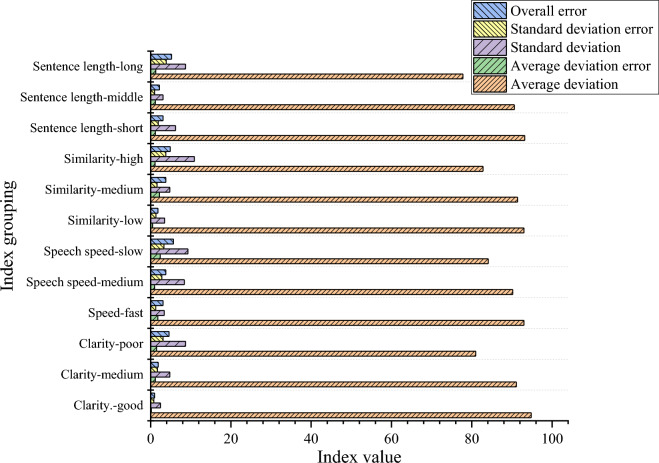
Descriptive statistics of grouped evaluation of different classroom discourse comprehensive scores.

Figure 3 presents the grouping and descriptive statistics on for speaking rate, speech intelligibility, average sentence length, and content similarity indicators of the classroom discourse of in three secondary school courses with the same name in the secondary school.

In the grouped online course evaluation, speech intelligibility is rated as “excellent” (97.9 points), “middle” (91.1 points), and “poor” (81 points). Speaking rate is rated as “fast” (93 points), “middle” (90 points), and “slow” (84 points). In comparison, content similarity is rated as “low” (93 points), “middle” (91.4 points), and “high” (82.8 points). Average sentence length is rated as “short” (93.2 points), “medium” (90.6 points), and “long” (77.8 points). The evaluation scores for different groups of indicators vary.

#### Analysis of variance

**Figure 4:**
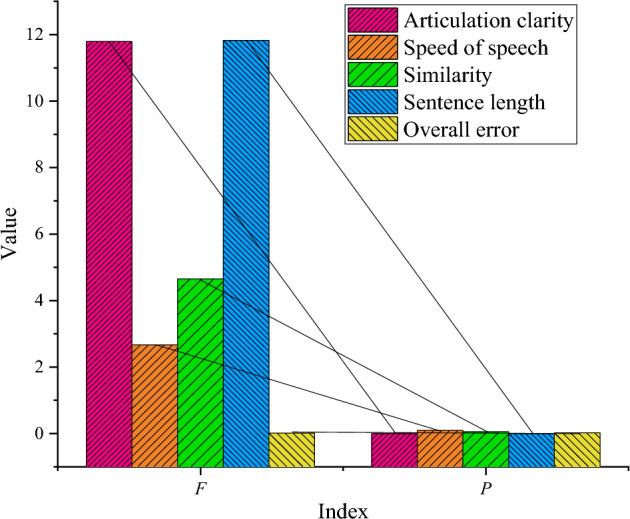
ANOVA of comprehensive scores of classroom discourse evaluation in different groups.

Figure 4 conducts an analysis of variance (ANOVA) to explore whether there are statistical differences in the classroom discourse evaluation scores of the four indicators between different groups.

In the ANOVA of the speech intelligibility dimension, *F* = 11.8 and *p* = 0.0009. In the ANOVA of the speaking rate, *F* = 2.67, and *p* = 0.093. In the ANOVA of the content similarity, *F* = 4.65, and *p* = 0.045. In the ANOVA of the average sentence length, *F* = 11.83, and *p* = 0.0008. The results indicate that the comprehensive scores of grouped evaluations among different indicators exhibit varying significance.

### Correlation analysis of the online classroom discourse indicators and course evaluation in secondary schools

**Figure 5:**
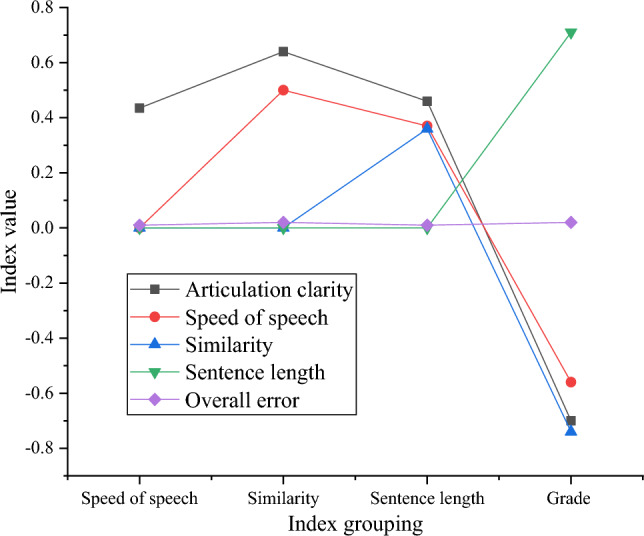
Correlation analysis between classroom discourse indicators and comprehensive scores of course evaluation.

Figure 5 illustrates the correlation analysis results between online classroom discourse indicators and comprehensive course evaluation scores in secondary schools.

In Fig. 5, a significant negative correlation is observed between speech intelligibility and the comprehensive score of online course evaluation, with a correlation coefficient of −0.71. The speaking rate is significantly negatively correlated with the comprehensive online course evaluation score, with a correlation coefficient of −0.56. The content similarity of classroom discourse is significantly negatively correlated with the comprehensive course evaluation score, showing a correlation coefficient of −0.74. The average sentence length of classroom discourse is significantly negatively correlated with the comprehensive online course evaluation score, with a correlation coefficient of −0.71.

### Regression analysis of classroom discourse indicators in secondary school online education on course evaluation

**Figure 6:**
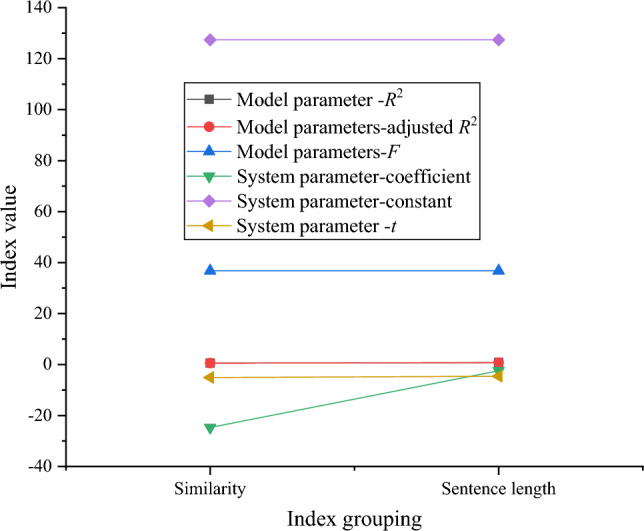
Results of stepwise multiple regression analysis of the impact of classroom discourse indicators on comprehensive course evaluation.

Figure 6 presents the results of stepwise multiple regression analysis examining the impact of classroom discourse indicators on learners’ course evaluation.

In Fig. 6, the model fitting equation is *y* = −24.74 (similarity) −4.64 (sentence length) + 127.44. The model fitting determination coefficient *R*^2^ = 0.78, the adjustment coefficient *R*^2^ = 0.76, and the model fitting and coefficient are highly significant. Among these, similarity emerges as the strongest explanatory variable, explaining the majority of the variation in the comprehensive course score, while sentence length contributes to a smaller portion of the variation in the comprehensive course score.

## Discussion

The experimental outcomes of this work demonstrate significant applications of deep learning and image recognition technologies in secondary education. Utilizing these advanced technologies enables a more comprehensive and objective assessment of online verbal communication among secondary school students, which is crucial for identifying and addressing teaching issues. Educators can practically use these results to promptly recognize and rectify communication challenges, thereby enhancing students’ positive experiences in online education. A key finding of this study is the understanding of the relationship between various verbal communication indicators and course evaluations, laying a theoretical foundation for personalized teaching support. This allows educators to adapt teaching methods flexibly based on students’ learning styles and needs, improving teaching’s specificity and effectiveness. Educators can better meet personalized learning needs through targeted teaching strategies, enhancing education’s overall effectiveness.

In practical applications, this work provides crucial data support for educational decision-makers, empowering them to make informed policy decisions and implement measures to enhance online course quality and effectiveness. It is recommended that educational decision-makers establish decision frameworks based on empirical data to drive improvements in the entire education system. Based on this, managerial recommendations include suggesting educational institutions incorporate deep learning and image recognition technologies into online education assessments to comprehensively understand teaching quality and student experiences. Educators can devise targeted teaching improvement strategies by identifying key verbal communication indicators, such as adjusting speech speed or enhancing speech comprehensibility, to elevate students’ learning experiences. Personalized learning experiences, especially in aspects like speech speed and content similarity, will aid students in better assimilating into the online learning environment, aligning more closely with subject interests and learning styles. Ultimately, this contributes to refining individual educators’ teaching methods and provides valuable insights for the entire education system’s development. In formulating online education policies, it is recommended that educational decision-makers fully leverage research results to promote evidence-based development. Understanding the relationship between verbal communication indicators and comprehensive course evaluations allows policymakers to precisely guide the direction of online education development, fostering overall improvements in educational standards. Emphasizing data-driven decision-making in the policy formulation process ensures the effectiveness and sustainability of policies, helping translate research findings into practical educational reforms and policy implementations.

## Conclusion

The implementation of the online “Gold Course Construction” plan initiated by the Ministry of Education, aimed at developing first-class online courses, is considered a crucial strategy for enhancing the quality of higher education in China, particularly in terms of talent training. Consequently, there has been a significant rise in the analysis and research on classroom discourse. This work builds upon previous research and utilizes AI to effectively mine and analyze teaching behaviors, specifically focusing on classroom discourse in online courses at the secondary school level. The primary emphasis is on constructing a CDA framework for online secondary school courses, providing the foundation for a dataset in subsequent experiments by integrating AI-driven data mining technology. The experimental findings highlight content similarity and average sentence length as the most influential indicators of classroom discourse, both falling under the strategic features category. Among these, content similarity is pivotal in learners’ online learning compared to average sentence length. It is essential to note that this work currently tests the effectiveness of CDA on only three types of English and Chinese courses in secondary schools. Future efforts will involve designing experiments to investigate whether similar characteristics and patterns exist in the classroom discourse of other disciplines. The ultimate goal is to offer methods and references for educators to enhance classroom discourse and strengthen teaching effectiveness.

### Supplementary Information

Supplementary Information.

## Data Availability

All data generated or analysed during this study are included in this published article [and its supplementary information files].
