# Supplementary material for: The use of deep learning integrating image recognition in language analysis technology in secondary school education
Source: Sci Rep. 2024 Feb 5;14:2888. doi: 10.1038/s41598-024-52592-5 (PMC10838925; doi:10.1038/s41598-024-52592-5)
Supplement: Supplementary file 1 — Supplementary Information. [file 41598_2024_52592_MOESM1_ESM.zip › data packet/Description for code.docx]

The above code pseudocode describes a process of online classroom discourse calculation, including the following main steps:

**1. ** Online course video acquisition and format conversion * *:** First, select major online course platforms, use web crawler programs to obtain teaching video resources, and convert these resources into data in audio and image formats.

**2. ** Convert classroom discourse and teaching content into text * *:** Next, convert audio resources into text by calling Alibaba Cloud intelligent speech recognition interface, and extract text information from image resources by using Baidu AI Cloud's universal scene text interface. This step converts audio and image data into text data, laying the foundation for subsequent analysis.

**3. ** Parameter calculation of classroom discourse indicators * *:** In this step, according to the CDA framework of middle school online courses, from the perspective of teaching objects, four classroom discourse indicators are designed, namely speech speed, phonetic clarity, average sentence length and text similarity. By calculating these indicators, unstructured text data can be converted into structured quantitative format, so as to analyze and evaluate the teaching quality of online classroom in the future.

The main program combines these steps together, and finally outputs the results of various indicators of classroom discourse, so that educators can better understand and improve the teaching effect of online classroom.
